# Social contacts and the locations in which they occur as risk factors for influenza infection

**DOI:** 10.1098/rspb.2014.0709

**Published:** 2014-08-22

**Authors:** Kin O. Kwok, Benjamin J. Cowling, Vivian W. I. Wei, Kendra M. Wu, Jonathan M. Read, Justin Lessler, Derek A. Cummings, J. S. Malik Peiris, Steven Riley

**Affiliations:** 1 School of Public Health, Li Ka Shing Faculty of Medicine, The University of Hong Kong, Hong Kong Special Administrative Region, People's Republic of China; 2 Centre for Influenza Research, School of Public Health, Li Ka Shing Faculty of Medicine, The University of Hong Kong, Hong Kong Special Administrative Region, People's Republic of China; 3 Faculty of Health and Life Sciences, Department of Epidemiology and Population Health, Institute of Infection and Global Health, University of Liverpool, Liverpool, UK; 4 Department of Epidemiology, Johns Hopkins Bloomberg School of Public Health, Baltimore, MD, USA; 5 MRC Centre for Outbreak Analysis and Modelling, Department of Infectious Disease Epidemiology, School of Public Health, Imperial College London, London, UK

**Keywords:** pandemic, influenza, contact patterns

## Abstract

The interaction of human social behaviour and transmission is an intriguing aspect of the life cycle of respiratory viral infections. Although age-specific mixing patterns are often assumed to be the key drivers of the age-specific heterogeneity in transmission, the association between social contacts and biologically confirmed infection has not previously been tested at the individual level. We administered a questionnaire to participants in a longitudinal cohort survey of influenza in which infection was defined by longitudinal paired serology. Using a variety of statistical approaches, we found overwhelming support for the inclusion of individual age in addition to contact variables when explaining odds of infection: the best model not including age explained only 15.7% of the deviance, whereas the best model with age explained 23.6%. However, within age groups, we did observe an association between contacts, locations and infection: median numbers of contacts (or locations) reported by those infected were higher than those from the uninfected group in every age group other than the youngest. Further, we found some support for the retention of location and contact variables in addition to age in our regression models, with excess odds of infection of approximately 10% per additional 10 contacts or one location. These results suggest that, although the relationship between age and incidence of respiratory infection at the level of the individual is not driven by self-reported social contacts, risk within an age group may be.

## 1. Introduction

The interaction of social behaviour and infectious disease transmission is a complex and fascinating process across many host–pathogen systems. Behaviours such as, social avoidance, mate choice, monogamy and group size, all influence the likelihood of transmission [1]. Even though snapshot data on any one aspect of behaviour does not capture the complex feedback within an entire population, good evidence of a causal link between behaviour type and infection has been reported many times, based on cross-sectional fieldwork. For example, badger *Meles meles* that move further and more frequently are more likely to be infected with bovine tuberculosis [2]. Among human pathogens, high rates of biting are associated with increased incidence of vector-borne infection [3] and numbers of sexual partners are predictive of an increased risk of HIV infection [4]. However, despite some suggestive evidence [5], the link between human behaviour and the transmission of respiratory viruses is less clear.

Social activities such as face-to-face conversations, skin-on-skin contact and the sharing of locations must, to some degree, influence an individual's risk of infection with a directly transmitted pathogen such as influenza [6–8]. However, the potential association between self-reported social contacts and the transmission of respiratory pathogens has not previously been tested empirically at the level of the individual. Those who report more frequent social contact should be at a higher risk of infection during an epidemic, baring other considerations such as immunity and differences in susceptibility.

The 2009 influenza pandemic, with substantial levels of infection in all but the eldest age groups, provided an ideal opportunity to investigate directly the correlation between self-reported social contacts, the locations at which those contacts occurred and infection with a common respiratory pathogen. Specifically, we wanted to test the hypothesis that the higher rates of infection observed in children relative to adults may be partly explained by differences in self-reported social contacts.

## 2. Material and methods

### (a) Fieldwork

We embedded an interviewer-led social-contact questionnaire (electronic supplementary material, text S1) within the 2009/2010 Hong Kong serological survey [9]. During telephone calls to schedule follow-up visits, participants were assigned a random day from days between the call and the visit. The nature of the questionnaire was described, and participants were asked to remember where they went and whom they met on their assigned day. Contact persons with whom subjects had face-to-face conversation or skin-on-skin contact were recorded. Details of contacts were also recorded, such as the location at which the contact occurred, the duration of the meeting and the approximate age of the contact. The questionnaire was administered face to face by the research team during the follow-up interview, and the forms were designed, so that supplemental sheets could be added easily. Hence, participants did not see a finite space that may have indicated subconsciously the desired number of contacts, nor the maximum number of contacts [10].

### (b) Infection outcome

Blood samples were taken at baseline and follow-up interviews. Infection was defined by a fourfold or greater rise in microneutralization (MN) titres against the 2009 pandemic influenza strain A(H1N1) A/California/4/2009 (with a final titre of 1 : 40 or greater) [9]. The endpoint of our MN assay was the highest serum dilution that suppressed cytopathogenic effect after 3 days incubation.

### (c) Base statistical model

The analyses we present here build on previously published statistical models of these infection outcomes for which the contact data used here were not available [9]. The best model in these previous analyses included the age of the participant as a linear term, the presence or absence of a child in the household and the district of residence. Although we did consider a smooth term for age [11], it was not supported once the presence or absence of child was also included.

### (d) Classifying exposure variables

Three groups of additional potentially explanatory variables were derived from the social-contact questionnaire and the standard questionnaire [9]: three key variables not related to the social-contact questionnaire (age of participant, presence or absence of a child in the household and district of residence; electronic supplementary material, table S1; as described above); 10 variables derived directly from specific questions about social contacts on the day of interest (e.g. number of contacts with duration greater than or equal to 10 min; electronic supplementary material, table S2) and three summary variables (average age of contacts, total minimum contact time and number of locations; electronic supplementary material, table S3).

### (e) Capturing group mixing

Individuals with large numbers of contacts or large numbers of contact locations often had a number of group meetings. Therefore, the questionnaire was administered so as to capture inherent uncertainty in the recall of groups. For example, when participants were not certain of the size of a group, they were encouraged to respond with a range for the number of people in a group. In addition, meetings of known size may have had different duration of contacts for subgroups or individuals. Because it was not feasible to break down fully every group contact (while maintaining a reasonable interview duration), interviewers recorded a single event with multiple durations. Therefore, for each contact variable (electronic supplementary material, tables S2 and S3), we inferred maximum and minimum values for each study participant from their responses. We used maximum, minimum and midpoint values (median of maximum and minimum) as potential explanatory variables in our regression analyses. We provide all individual-level data used for these analyses in the electronic supplementary material, dataset S1.

### (f) Hypothesis-driven best subset search of model space

We designed our statistical analyses to build on previous work in which age, district of residence and the presence or absence of a child in the household had all been found to be important determinants of the odds of infection. In moving on to examine self-reported social contacts, our primary hypothesis was that age effects in our previous work could be explained by some reasonable combination of self-reported social behaviour.

Every question on the contact questionnaire was asked because it may have captured some aspect of that behaviour. However, not all the questions were independent of each other, either numerically or conceptually. In addition, we were limited to approximately 80 positive outcomes and wanted to stay within the safe ratio of one parameter for each 10 positives for logistic regression. Therefore, we defined a set of 1408 models based on our own perception of the relationship between alternate variables (electronic supplementary material, section Material and methods). We fitted those models to the data as they were and then again with the addition of a linear age term and compared the results using standard information criteria.

### (g) Hypothesis-agnostic group lasso search of model space

Although our preference was to use a hypothesis-driven approach, best subset regression has been criticized because it can be biased towards lower *p*-values and higher effect sizes [12]. Therefore, we also analysed our data without pre-judging the likely degree of collinearity in our exposure variables but within a framework with an explicit penalty for additional parameters. We used group lasso logistic regression [13] with *λ* set to 4 (approximately natural log of number of groups). In order to explore the sensitivity of our model results to outliers in our data and to obtain confidence intervals for our non-likelihood method, we conducted a number of analyses on bootstrapped variants of our data. For each bootstrap dataset, we drew the same number of individual records (participants) from our data with replacement. Confidence intervals for bootstrap runs were the middle 95% point estimates.

## 3. Results

### (a) Study population

The parent study was a community-based serological survey of households in Hong Kong, recruited using random digit dialling [9]. The first baseline blood sample was taken on 4 July 2009 and the last on 19 September 2009, whereas the first follow-up sample was taken on 11 November 2009 and the last taken on 6 February 2010. The 770 participants who provided paired sera in the parent study also provided their contact diaries in their first follow-up visit (of which eight were excluded because their contact diaries were not sufficiently complete). Of these eight, only a single 9 year old was infected. The ages of the remaining seven uninfected excluded individuals ranged from 8 to 73 years. The cohort for the current study was made up of 762 individuals of whom 77 were defined to have been infected (electronic supplementary material, dataset S1).

### (b) Contacts and locations

**Figure 1.**
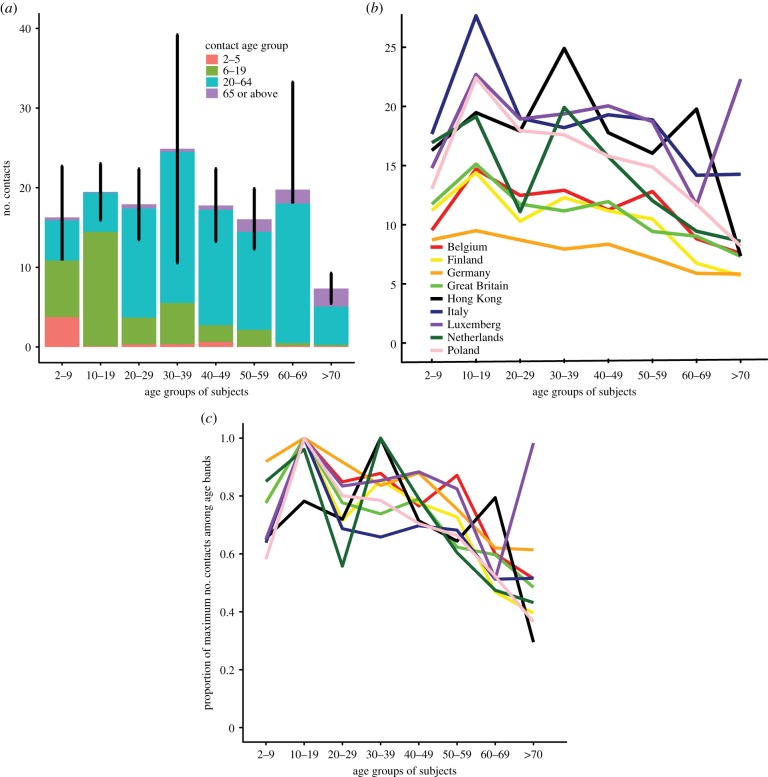
Age-specific contact patterns. (*a*) Distribution of self-reported contacts by age group for the present study. Values are the average per participant of the midpoint between reported maximum and minimum. Vertical black lines indicate binomial confidence bounds for the number of contacts across all four contact age classes for each participant age group. (*b*) Comparison of total number of contacts per age group for eight European countries, as reported in the PolyMod study [8]. PolyMod reported both participants and contacts in 5 year age groups starting with 0–4 years. Therefore, other than for the lowest age class, we used linear interpolation to generate consistent age boundaries from our less detailed data. For the lowest age class of participants, we report a range of 2–4 years, because our youngest participant was 2 years old. We assumed that PolyMod recruited few 0 and 1 year olds, so we did not interpolate for the lowest participant age class. We did not ask participants to report their contacts in 5 year age groups. (*c*) Also compares total contacts between the present study and the PolyMod study. However, in (*c*), although the relative amplitude for each age group for individual populations has been preserved, the amplitude has been rescaled, so that the maximum value for each country for any age group is 1.

The total of the number of contacts reported by all 762 participants was between 13 730 (sum of minimum values) and 14 163 (sum of maximum values), giving a range of 18.0–18.6 contacts per person. Adults between 30 and 39 years made more contacts than did other age groups in our study (figure 1*a*), even if subjects with a number of contacts more than 100 were excluded. Young children (2–9 years) and adults between 50 and 59 years made slightly fewer contacts, whereas adults over 70 years of age made considerably fewer contacts. Contacts were associative by age: the greatest number of contacts in the 2–5 year range were made by participants in the first age group; the greatest number of contacts in the 6–19 year range were made by participants in the 10–19 year group and the greatest number of contacts over age 65 were made by participants in our study aged 70 or older.

These data on age-specific contact patterns are consistent with the range of patterns reported by PolyMod, a study of potentially infectious social contacts in European populations [8]. Participants in the present study reported similar absolute numbers of contacts as those from European countries at the higher end of the PolyMod range, such as Italy and Luxembourg. The age-specific trends reported here (rescaled to have the same maximum amplitude; electronic supplementary material, figure S1*b*) are similar to those reported for most of the European countries such as the Great Britain, Italy and Poland in PolyMod, other than that adults between 30 and 39 years had proportionately more contacts than in other countries. Most countries in the European study showed consistently decreasing numbers of contacts with increasing age.

**Figure 2.**
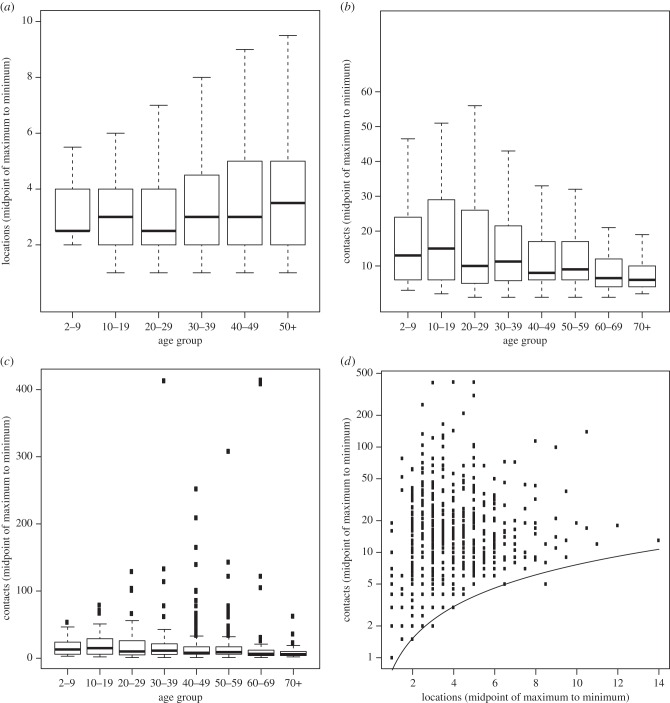
Comparison between locations and contacts. (*a*) Box and whisker plot [14] for total number of reported locations (midpoint of maximum and minimum) for participant age groups. (*b*) Box and whisker plot for total number of reported contacts (midpoint of maximum and minimum) for participant age groups. Note that participants with number of reported contacts >100 are not plotted in this chart. (*c*) This figure is the reproduction of (*b*) except that this represents the subjects with number of reported contacts >100. (*d*) Scatter plot of total number of reported locations (midpoint of maximum and minimum) and total number of reported contacts (midpoint of maximum and minimum). Above and to the left of the *y* = *x* line, most of the participants reported at least as many contacts as locations.

The total number of locations at which potentially infectious contacts were made by participants in our study was between 2335 (sum of minimum values) and 3007 (sum of maximum values), giving a range of 3.06–3.95 locations per person. This range was substantially wider, in proportionate terms, than the range observed for contacts. Variation in the number of locations per participant, as measured by interquartile range, increased consistently with age until a decrease for the eldest age group (figure 2*a*). By contrast, the variability in the number of contacts was highest for participants between 10 and 29 years of age and then fell away to higher ages (figure 2*b,c*).

Numbers of contacts were only weakly correlated with numbers of locations, once the number of contacts were adjusted to reflect the fact that almost every location included at least a single additional contact (although not all did, as the same single contact was occasionally met in multiple locations). The Spearman rank correlation coefficient between contacts and locations was 0.44 (figure 2*d*). However, given that a location can be reported only if at least one contact occurs, we also calculated the correlation between numbers of contacts minus number of locations with number of locations, which dropped to 0.18. Although both values are significantly different from 0 (*p*-values both < 0.001), these levels of correlation were lower than might be expected and justified the inclusion of both contact and location variables in the same statistical model.

### (c) Age, contact variables and infection status

Based on the raw data, both age and number of contacts were correlated with infection status (table 1). However, using the best subset approach (see Materials and methods), we showed that models including self-reported contact and location variables but without participant age were not able to describe these individual-level infection data as well as regression models that included age as a covariate (electronic supplementary material, figure S3 and dataset S2). The best model without age (electronic supplementary material, table S4) had an Akaike information criteria (AIC) of 439, whereas the worst model with participant age had an AIC of 411 (electronic supplementary material, dataset S2). The best model not including age explained only 15.7% of the deviance, whereas the best model with age explained 23.6% of deviance.

**Table 1.** The number and percentages of subjects who were seroconverted by age group and minimum number of contacts.

	minimum number of contacts								
age group	1–2 (*n* = 54)	3–4 (*n* = 94)	5–6 (*n* = 130)	7–8 (*n* = 94)	9–10 (*n* = 57)	11–19 (*n* = 160)	20–100 (*n* = 155)	100+ (*n* = 18)	% of positive
3–9 (*n* = 21)	0	2	4	0	1	1	3	0	52.4
10–19 (*n* = 89)	1	4	3	1	2	6	15	0	36.0
20–29 (*n* = 85)	0	0	1	0	2	1	4	0	9.4
30–39 (*n* = 60)	0	0	0	2	0	2	0	1	8.3
40–49 (*n* = 177)	1	0	1	3	1	5	2	1	7.9
50–59 (*n* = 202)	1	0	2	0	0	3	0	0	3.0
60–69 (*n* = 85)	0	0	1	0	0	0	0	0	1.2
70+ (*n* = 43)	0	0	0	0	0	0	0	0	0
% of positive	5.6	6.4	9.2	6.4	10.5	11.3	15.5	11.1	

### (d) Social contacts within age groups

**Figure 3.**
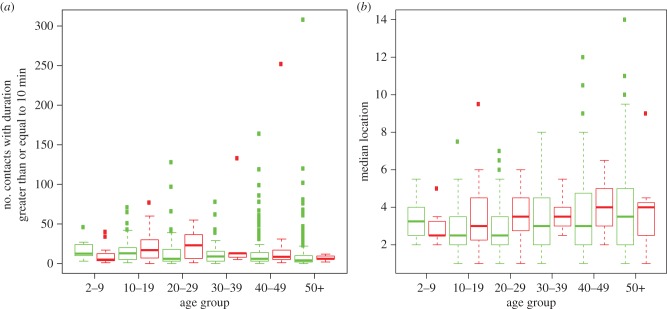
Infection, age, contacts and locations. (*a*) Distribution of number of contacts with duration greater than or equal to 10 min per age group and infection status. Inset same as main chart but with wider *y*-axis showing all outliers. (*b*) Distributions of numbers of locations per age group and infection status. In both parts: green denotes not infected and red infected; features follow the usual convention for box and whisker plots [14].

The contribution of contacts and locations to the risk of infection can be seen explicitly by separating age groups into those who were infected and those who were not (figure 3*a,b* and electronic supplementary material, figure S2). Other than the youngest age group, the median number of contacts with duration greater than or equal to 10 min for those infected in an age group is always greater than for those not infected in an age group (Wilcoxon test, *p*-value < 10^−4^, for non-paired comparison of infected and uninfected). Similarly, again with the exception of the youngest age group, the median number of locations for those infected is greater than for those who were not. However, likely due to the lower typical value for number of locations, the difference between the infected and uninfected subgroups for location was not statistically significant (*p*-value = 0.45). The reversal of the trend in the youngest age group may reflect lower accuracy of reported contacts.

### (e) Contacts and locations

We found some support for models of infection that included both contact and location variables in addition to age and other baseline variables, over and above those that included only age and other baseline variables (table 2). The best of these models included the number of locations and the number of contacts greater than or equal to 60 min and had an AIC of 399.3 (compared with an AIC of 402.3 for the baseline model). The estimates of odds ratios of infection for number of contacts with duration greater than or equal to 60 min and number of locations were 1.09 and 1.15, respectively. In other words, an increase in one location visited and one long contact increased the odds of infection by 9% and 15%, respectively. In addition, the best 27 models overall included contact, location and baseline variables. Out of models that included only contact and baseline variables, the best model included the number of contacts with duration greater than or equal to 10 min.

**Table 2.** Regression model results from best subset analysis and group lasso regression.

	best subset								group lasso	
	baseline model		best model with location variable only		best model with contact variables		best model with contact and location variables		estimate	95% CI^b^
age	0.942	(0.926–0.957)	0.939	(0.923–0.955)	0.942	(0.926–0.958)	0.939	(0.923–0.956)	0.945	(0.928–0.960)
district (ref Hong Kong Island)										
Kowloon East	1.06	(0.348–3.24)	1.03	(0.338–3.16)	1.06	(0.346–3.27)	1.03	(0.335–3.17)	1.00	(0.580–1.70)
Kowloon West	2.58	(0.915–7.29)	2.61	(0.918–7.41)	2.58	(0.905–7.37)	2.57	(0.898–7.38)	1.68	(1.00–3.96)
New Territories East	2.47	(1.04–5.87)	2.45	(1.03–5.83)	2.5	(1.05–5.97)	2.44	(1.02–5.84)	1.71	(1.11–3.35)
New Territories West	1.51	(0.584–3.89)	1.47	(0.567–3.81)	1.54	(0.592–4.01)	1.50	(0.574–3.91)	1.23	(0.828–2.23)
presence of child	2.49	(1.31–4.73)	2.47	(1.3–4.71)	2.49	(1.31–4.74)	2.46	(1.29–4.69)	2.06	(1.21–3.46)
number of locations (mean of maximum and minimum, per location)	—		1.16	(0.998–1.34)	—		1.15	(0.989–1.34)	1.08	(1.00–1.18)
contacts greater than 10 min (minimum, per 10 contacts)	—		—		1.10	(1.01–1.19)	—		1.03	(1.01–1.22)
contacts greater than 60 min (minimum, per 10 contacts)	—		—		—		1.09	(1.00–1.19)	1.04	(1.00–1.16)
AIC	402.3		400.7		400.3		399.3			
goodness-of-fit *p*-value^a^	0.38		0.31		0.25		0.28			
% of deviance explained	22.2		22.9		23.0		23.6			

^a^Based on the le Cessie–van Houwelingen–Copas–Hosmer unweighted sum of squares test for global goodness of fit [15], calculated using the lrm package in R.

^b^Middle 95% of values from bootstrap refits in which the parameter was retained in the final model.

The magnitude and significance of the contact and location variables were consistent. The increase in odds of infection per location was approximately 15% but only of borderline significance. The increase in odds of infection per 10 contacts was approximately 10%, and was usually significant (at the 5% level; the electronic supplementary material, dataset S2). The values of the other parameters were consistent across the four best models of each type (table 2): all were equal up to two significant figures other than the district parameter for New Territories East in the best model with contact and location variables. All four of the best models were consistent with the data using standard goodness-of-fit tests for logistic regression models [16].

### (f) Sensitivity analysis

Of the 762 participants in this study, 616 characterized the day for which they reported contacts as being a typical day. We recalculated our regression model results for this subset of the cohort (electronic supplementary material, table S5), finding very similar results to those presented above. For the typical day set of best models, the two different contact variables (greater than 10 min and greater than 60 min) were replaced by a single variable: number of contacts greater than or equal to 30 min. In addition, the location variable in the best model with location and not contacts became borderline significant, rather than borderline not significant (to the 5% level).

We also examined association between these exposure data and infection using a hypotheses-agnostic lasso regression model [13] (table 2). In general, the lasso regression results are supportive of the best subset results. Although the point estimates for the strength of effect of contact variables (number of locations and number of contacts with duration greater than 60 min) are reduced (as would be expected [12]), they remain statistically significant in models selected using group lasso regression. Interestingly, some social behaviour parameters other than those retained in our best subset analysis are retained more frequently in a bootstrap analysis of the group lasso models (electronic supplementary material, table S6). However, the bootstrap confidence intervals for these tended not to be significant.

## 4. Discussion

We embedded a questionnaire within a longitudinal serological cohort survey to test the strength of association between self-reported contacts and odds of influenza virus infection. From the questionnaire, we extracted variables related to individual contacts and to the locations at which contacts were made. Numbers of locations and contacts reported by individuals were only weakly correlated. Among a large set of logistic regression models, each with serologically confirmed influenza virus infection as the outcome, we found overwhelming support for models that used participant age explicitly, over models that used only contact variables: contact behaviour alone at the level of the individual did not explain age-specific odds of infection. Within age groups, both number of locations and numbers of contacts increased odds of infection (approx. 10% per 10 contacts or per location; models that did include these terms received similar support to those that did not). These age-specific patterns of infection could be seen explicitly in the data: other than the youngest age group, the median number of contacts or locations was always higher in those infected (in a given age group) than in those not infected.

It is not possible or desirable to consider every possible statistical model. There may be spline- or interaction-based logistic models that are better able to reproduce the patterns seen in these data. However, so far as is ever possible, we have sought in this initial report to describe the key features of the data and the relationship between our data and a large set of models in which we had an interest prior to describing the data. While we acknowledge that some of the parameter estimates may be considered only marginally significant, we achieved good consistency over a variety of regression modelling techniques. It is noteworthy that the parameters retained in our best subset analysis were always significant in the lasso regression bootstrap analysis. This does point towards a subset of influential data points. However, that is not unexpected given the strong right-skew commonly observed in the distribution of social-contact variables. All individual-level data are provided, so that others can continue these lines of investigation (electronic supplementary material, dataset S1).

Although our results are based only on a single population, we believe that, as the first robust investigation of the association between self-reported social contacts and biologically confirmed respiratory infection [6], these results represent a useful addition to population-level evidence [5,17,18]. The requirement for age in our regression models indicates clearly that factors other than self-reported contacts of individuals—as recorded here—drove individual odds of infection.

There are a number of explanations for the inability of social contacts to account for age-specific infection patterns in our data using these modelling approaches. First, the way that one age group makes contacts and reports them may be very different from another age group. Direct observation- or proximity-based studies may be able to tease out these differences [6]. In addition, it is possible that there was a unique profile of susceptibility to the 2009 pandemic strain that will not be present for other influenza pandemics. If we had studied a population of fully immunologically naive individuals across all age groups, we may well have seen a different relationship.

We chose to present these data initially using traditional epidemiological approaches for primary analysis and we have not fitted any mechanistic transmission models. A secondary analysis used a stratified final size model (with arbitrary resolution in age and numbers of contacts) to capture the contact structure of the population [19] and was able to show that the average behaviour of an age group, as measured by self-reported social contacts, was an important determinant of infection risk.

Methods of measuring potentially infectious contacts are themselves [6] a topic of active investigation. Here, we chose a retrospective interviewer-led questionnaire approach. Participants were informed in advance, by phone, of the nature of the discussion, but they were not given a diary. Although there are unavoidable issues with recall-bias, this design has many advantages over alternatives: it does not directly invade privacy and hence change behaviour; it is not overly sensitive to the specific design of the form (because the participant did not see the form), contacts within large buildings are as likely to be recorded as those in the open air, and there is no need to recruit potential contacts into the study, so that they can wear study equipment. Perhaps most importantly, we are measuring what participants themselves consider to be a contact. Therefore, robust associations between self-reported contacts and infection outcomes generate, by definition, evidence that could be actionable by individuals: people may not note how many other people they come within 1 m of in a given day, but they do know the number of people they talk to and shake hands with.

We asked only about a single day immediately prior to the follow-up interview. Given that antibody titres take up to three weeks to rise after infection [20], it is unlikely that we asked about the actual day that people were infected. Therefore, we implicitly make the assumption that variation between individuals in their social behaviour for both contacts and locations was stationary in time and, by asking about a single day, we obtained a representative snapshot of that behaviour. It is possible that self-reported contacts—recorded in exactly the same manner we did here—for the actual day of exposure may explain variability in the odds of infection with greater accuracy. However, our sensitivity analysis using the participant's characterization of the day in question as being typical or atypical suggests otherwise. Therefore, our results motivate two specific refinements of this questionnaire-based approach. First, prospective repeat questionnaire studies, without biological outcome data, would help characterize the degree to which responses from the same individual remain constant over time [6]. Second, a case–control approach could be used to obtain biologically confirmed infection outcomes and social-contact data immediately prior to the onset of symptoms.

## Supplementary Material

Electronic Appendix

## Supplementary Material

Dataset S1

## Supplementary Material

Dataset S2

## Notes

All study protocols and instruments were approved by the institutional review boards of the University of Hong Kong. Written informed consent was sought from each participant aged 18 or above. Written proxy consent was sought from the head of household of all participants aged 17 or below. In addition, the written assent to participate was asked from participants of aged 7–17.
