# Supplementary material for: Social contacts and the locations in which they occur as risk factors for influenza infection
Source: Proc Biol Sci. 2014 Aug 22;281(1789):20140709. doi: 10.1098/rspb.2014.0709 (PMC4100506; doi:10.1098/rspb.2014.0709)
Supplement: Electronic Appendix [file rspb20140709supp1.doc]

**Electronic supplementary material**

**Social contacts and the locations in which they occur as risk factors for influenza infection**

Kin O. Kwok^1^, Benjamin J. Cowling^1^, Vivian W. I. Wei^1^, Kendra M. Wu^1^, Jonathan M. Read^2^, Justin Lessler^3^, Derek A. Cummings^3^, J.S. Malik Peiris^4^, and Steven Riley*^1,5^

1. School of Public Health, Li Ka Shing Faculty of Medicine, The University of Hong Kong, Hong Kong Special Administrative Region, People’s Republic of China.
2. Department of Epidemiology and Population Health, Institute of Infection and Global Health, Faculty of Health and Life Sciences, University of Liverpool, Liverpool, UK.
3. Department of Epidemiology, Johns Hopkins Bloomberg School of Public Health, Baltimore, USA.
4. Centre for Influenza Research, School of Public Health, Li Ka Shing Faculty of Medicine, The University of Hong Kong, Hong Kong Special Administrative Region, People’s Republic of China.
5. MRC Centre for Outbreak Analysis and Modelling, Department of Infectious Disease Epidemiology, School of Public Health, Imperial College London, United Kingdom.

*Email: [steven.riley@imperial.ac.uk](mailto:steven.riley@imperial.ac.uk).

Contents:

Supplementary Material and methods

Supplementary tables S1-S6

Supplementary figures

Text S1

Data S1-S2

References

**SUPPLEMENTARY MATERIAL AND METHODS**

**Hypothesis-driven best subsets regression**

We considered 1,408 logistic regression models (Dataset S2) with infection as the outcome and different combinations of the 39 potentially explanatory social contact variables. Our rationale for searching through model space was influenced by the following factors: we wanted to keep the total number of parameters in any model less than 8, so as to remain close to the 1 parameter per 10 positive cases; we did not want to introduce obvious colinearity by using alternate forms of the same variable (e.g. maximum and minimum in the same model); nor did we want to consider models that included strongly dependent variables (e.g. number of contacts greater than 10 minutes and number of contacts greater than 60 minutes). All models included terms for the presence or absence of a child in the household and district of residence (Table S1 blocks 2 and 3) and were analysed using the base function glm in the R statistical package 2.15 .

Because our primary objective was to see how well self-reported contacts accounted for the strong age-association with risk of infection we have observed previously , half the models included a linear term for the age of the participant and half did not. Within the 1,408 models in these two sets, we made the following groups; baseline (number of models, n=1), baseline plus location variables only (n=3), baseline plus contact variables only (n=351), and baseline plus location plus contact variables (n=351x3=1,053). We used three variables related to location: maximum number of locations, minimum number of locations and the mid-point of maximum and minimum (Table S3 block 3). We used two types of variables related to self-reported contacts: direct measures (Table S2) and summary measures (Table S3 blocks 1 and 2). The 351 members of the contact variables set was made up of: the 27 combinations of choosing exactly one from all direct measures and summary measures; the 126 combinations of choosing exactly 1 direct measure and one summary measure; the 9 combinations of choosing one summary age measure (Table S3 block 1) and one summary time measure (Table S3 block 2); and the 189 combinations of choosing exactly one direct measure, one summary age measure, and one summary time measure.

**Hypothesis-agnostic group lasso regression**

Although our preference was to use a hypothesis-driven approach, best subset regression has been criticised because it can be biased towards lower p-values and higher effect sizes . Therefore, we also analysed our data without pre-judging the likely degree of colinearity in our exposure variables but within a framework with an explicit penalty for additional parameters. We used group lasso logistic regression with $\lambda$ set to 4 (~ natural log of number of groups). In order to explore the sensitivity of our model results to outliers in our data and to obtain confidence intervals for our non-likelihood method, we conducted a number of analyses on bootstrapped variants of our data. For each bootstrap dataset, we drew the same number of individual records (participants) from our data with replacement. Confidence intervals for bootstrap runs were the middle 95% point estimates.

**SUPPLEMENTARY TABLES**

**Table S1** Baseline potentially explanatory variables for logistic regression, not related to social contacts or locations.

**Table S2**  Potentially explanatory variables describing numbers of self-reported contacts.

**Table S3** Potentially explanatory variables that summarise characteristics of contacts and locations.

**Table S4.** Parameter estimates for the three best models that did not include age but did include: baseline variables only, baseline plus location variables, baseline plus contact variables only and baseline plus contact plus location variables.

**Table S5.** Parameter estimates for the three best models from data characterized the day for which the subjects reported contacts as being a typical day: baseline variables only, baseline plus location variables, baseline plus contact variables only and baseline plus contact plus location variables.

**Table S6.** Results of the grouped lasso logistic regression compared with results from hypothesis-driven best subsets approach.

|  | Estimate | 95 % CI | % occurrence in  1000 bootstrapped  models |
| --- | --- | --- | --- |
| Age | 0.944 | (0.928-0.960) | 100 |
| District (ref Hong Kong Island) |  |  | 99.7 |
| Kowloon East | 1.05 | (0.580-1.70) |  |
| Kowloon West | 2.05 | (1.00-3.96) |  |
| New Territories East | 1.95 | (1.11-3.35) |  |
| New Territories West | 1.35 | (0.828-2.23) |  |
| Presence of child | 2.13 | (1.21-3.46) | 98.3 |
| Minimum number of people with whom the  participant had skin-to-skin contacts | 0.985 | (0.963-0.999) | 62.6 |
| Typical Day | 1.26 | (0.827-1.98) | 62.3 |
| Maximum average (per participant) minimum  duration of contact | 1.01 | (1.00-1.02) | 60.1 |
| Average age of (minimum) contacts | 0.980 | (0.946-0.998) | 49.5 |
| Minimum number of location | 1.13 | (1.00-1.32) | 46.7 |
| Maximum number of locations | 1.08 | (1.00-1.18) | 45.0 |
| Maximum number of people with whom the  participant had face-to-face contact whom the  participant estimated to be 5 years old or younger | 1.05 | (1.00-1.31) | 40.5 |
| Number of locations (mean of maximum and minimum, per location) | 1.09 | (1.00-1.18) | 39.2 |
| Contacts greater than 10 minutes (minimum, per 10 contacts) | 1.07 | (1.01-1.22) | 32.2 |
| Minimum number of people with whom the  participant had face-to-face contact whom the  participant estimated to be 19 years old or younger | 1.01 | (1.00-1.02) | 30.9 |
| Minimum number of people with whom the  participant had face-to-face contact whom the  participant estimated to be 5 years old or younger | 1.03 | (0.981-1.09) | 29.1 |
| Contacts greater than 60 minutes (minimum, per 10 contacts) | 1.07 | (1.00-1.16) | 28.7 |

**SUPPLEMENTARY FIGURES**

**Fig. S1A.** **Comparison between this study and PolyMod .** This chart is the reproduction of figure 1B (subjects with the number of contacts >100 will be excluded).

**Fig. S1B.** **Rescaled comparison between this study and PolyMod .** This chart is a variant of Figure 1C. The relative amplitude for each age group for individual populations has been preserved but the amplitude has been rescaled so that the maximum value for each country for any age group is 1 (subjects with the number of contacts >100 will be excluded).

**Fig. S2. Distribution of numbers of contacts greater than or equal to 10 minutes per age group and infection status with outliers.** This figure is the reproduction of figure 3A without outliers.

**Fig. S3. Logistic regression model comparison.** Frequency distribution of 1,408 regression models for 5-unit bins of Akiake Information Criterion (AIC). Exactly half the models are on the left hand side of the chart and include a linear term for continuous age of the participant. The other half, on the right hand side of the chart, do not include the linear age term. Within each half (n=1,408) models are further categorized into; baseline (n=1, red), baseline plus contact terms only (n=351, green), baseline plus location terms only (n=3, purple), and baseline plus contact and location terms (n=1,053, blue). A null model, in which the probability of infection is equal to the number of infections divided by the total number of participants, has an AIC of 501 on this scale (not shown).

**Text S1** Social contact questionnaire (English).

**Dataset S1 and S2 are attached as csv format.**

**Dataset S1.** **Individual-level data.** Tables S1, S2 and S3 act as a data dictionary for most of the fields in these data (all exposure variables). Fields not in Tables S1, S2 or S3 are; "ffold" the column heading for infected (0 not infected, 1 infected), and "id" is the unique individual identifier.

**Dataset S2.** 1408 Regression models and each row represents one model. The first row shows the name of the exposure variables.

References

1. 2010 R.D.C.T. 2010 *R: A language and environment for statistical computing*. Vienna, R Foundation for Statistical Computing.

2. Wilson P.W., D&apos;Agostino R.B., Levy D., Belanger A.M., Silbershatz H., Kannel W.B. 1998 Prediction of coronary heart disease using risk factor categories. *Circulation* **97**(18), 1837-1847.

3. Riley S., Kwok K.O., Wu K.M., Ning D.Y., Cowling B.J., Wu J.T., Ho L.-M., Tsang T., Lo S.-V., Chu D.K.W., et al. 2011 Epidemiological characteristics of 2009 (H1N1) pandemic influenza based on paired sera from a longitudinal community cohort study. *PLoS Med* **8**(6), e1000442. (doi:10.1371/journal.pmed.1000442).

4. Harrell F.E. 2001 *Regression Modeling Strategies*, Springer Verlag; 568 p.

5. Meier L., Van De Geer S., Bühlmann P. 2008 The group lasso for logistic regression. *Journal of the Royal Statistical Society: Series B (Statistical Methodology)* **70**(1), 53-71.

6. Mossong J., Hens N., Jit M., Beutels P., Auranen K., Mikolajczyk R., Massari M., Salmaso S., Tomba G.S., Wallinga J., et al. 2008 Social contacts and mixing patterns relevant to the spread of infectious diseases. *PLoS Med* **5**(3), e74. (doi:10.1371/journal.pmed.0050074).
